# Treatment adherence among new triptan users: a 2-year cohort study in Taiwan

**DOI:** 10.1186/1129-2377-15-48

**Published:** 2014-08-12

**Authors:** Ting-Bin Chen, Yung-Tai Chen, Jong-Ling Fuh, Chao-Hsiun Tang, Shuu-Jiun Wang

**Affiliations:** 1 Department of Neurology, Neurological Institute, Taipei Veterans General Hospital, No. 201, Sec. 2, Shipai Rd., Beitou District, Taipei 11217, Taiwan; 2 Faculty of Medicine, National Yang-Ming University School of Medicine, Taipei, Taiwan; 3 Department of Nephrology, Institute of Internal Medicine, Taipei City Hospital Heping Fuyou Branch, Taipei, Taiwan; 4 Institute of Brain Science, National Yang-Ming University, Taipei, Taiwan; 5 School of Health Care Administration, Taipei Medical University, Taipei, Taiwan

**Keywords:** Triptan persistence, Migraine medication adherence, Refill patterns, Taiwan

## Abstract

**Background:**

The persistence of triptan use among newly prescribed users is low in the United States and European countries. However, triptan refill patterns in Asian primary care practices have not been well described.

**Methods:**

Data from the National Health Insurance Research Database in Taiwan were used to conduct a retrospective cohort analysis from 2005 to 2008. All participants were followed for 2 years after receiving a new triptan prescription. Refill and 2-year retention rates of newly prescribed triptans were calculated, and predictors of the first triptan refill and 2-year retention were analyzed.

**Results:**

Of the 13,951 participants with a new triptan prescription (99.9% sumatriptan), 67.4% were prescribed by a neurologist, 67.4% were prescribed at least one prophylactic agent for migraine. Of them, 34.3% adhered to the newly prescribed triptan at the first refill, 0.01% switched to another triptan, and 40.9% switched to a non-triptan acute migraine medication. The 2-year retention rate was 4.0%. The frequency of headache-related neurologic visits for 1 year before the index date, first prescription of triptan or other acute medications, first triptan prescription by a neurologist, and prophylactic use were associated with higher first refill rates. The frequency of headache-related neurologic visits 1 year before the index date and first triptan prescription by a neurologist were related to higher 2-year retention rates. Diabetes mellitus and first triptan prescription at a local medical clinic were associated with reduced probability of continued triptan use at the first refill and 2 years.

**Conclusions:**

Similar to Western societies, the refill and 2-year retention rates were low in new users of triptans. Frequency of neurologic visits and triptan prescription by a neurologist were significant predictors of adherence.

## Background

Migraine is a common, chronic, and disabling disorder of neurovascular etiology, characterized by recurrent episodic attacks and a variable presentation among patients. In addition to its impact on the quality of life of afflicted individuals, migraine imposes a substantial financial burden on healthcare systems and employers [1]. The prevalence of migraine in Taiwan is 9.1% among people 15 years and older, with a prevalence of 14.4% and 4.5% in women and men, respectively [2]. Because many cases of migraine are chronic and intermittent, it is recommended that patients undergo correct pharmacologic management and retain the prescribed treatment to avert or relieve debilitating pain, prevent escalation to acute medications, and improve day-to-day functioning [3]. Although preventive treatment can reduce disability by decreasing the number and severity of attacks, many patients still required acute abortive treatments [4].

The treatment of acute migraine is challenging because of the high nonresponse rates to medications. Moreover, it is difficult to predict individual response to a specific agent or dose [5]. In the primary care setting, different panels and guidelines recommend various medications [6-9]. Categories include nonspecific and specific treatments. Nonspecific treatments are those effective for any pain disorder and include non-steroidal anti-inflammatory drugs (NSAIDs), combination analgesics, opioids, neuroleptics/antiemetics, and corticosteroids. Specific therapies, such as ergotamine-containing compounds, dihydroergotamine, and triptans, are effective only for migraine.

For many migraine patients, triptan therapy provides complete pain relief of some attacks but not of others. Triptan therapy is more likely to achieve complete pain relief if administered early. In the United States and European countries, triptans are recommended as a first-line therapy for moderate-to-severe migraine [10,7]. According to the regulations for triptan use proposed by the Taiwan National Health Insurance (NHI), triptans can be prescribed only when the headache characteristics fulfill the first edition of the diagnostic criteria of migraine by the International Headache Society (1988) [11], and the headache does not respond to other acute medications. Only two kinds of triptans, sumatriptan and rizatriptan, are available in Taiwan and have been provided to the public by the NHI since 1999 and 2008, respectively. The maximum recommended dosage per month of oral sumatriptan is 400 mg, of intranasal sumatriptan is four doses of 20 mg, and of oral rizatriptan is 40 mg.

Based on the 2009 National Ambulatory Care Survey in the United States, over 80% of prescribed specific antimigraine drugs were triptans. Of these, sumatriptan accounted for almost half of all triptan prescriptions; rizatriptan and eletriptan together accounted for about a third [12]. Despite the widely reported efficacy and tolerability of triptans, several studies showed that the discontinuation of triptans was high [13-15]. According to a US pharmacy claims database and European nationwide practice databases, more than 50% of newly prescribed triptan users never refilled their first triptan prescriptions during a 2-year follow-up [16,17]. Moreover, only ~10% of newly prescribed triptan users continued to use the same triptan that was originally prescribed in a 2-year follow-up period [16,17].

However, there have not been any reports regarding adherence to, discontinuation of, and usage patterns of migraine abortive medications in Asian countries. To address this issue, we conducted a nationwide population-based cohort study, with a similar design to those conducted in the United States and Europe, to investigate prescription refill patterns and predictors of adherence among users of newly prescribed triptans, on the basis of the National Health Insurance Research Database (NHIRD) in Taiwan.

## Methods

### Data source

A population-based retrospective cohort study was performed with data from the Taiwan NHIRD. Since 1995, Taiwan’s National Health Insurance (NHI) program has integrated all of the public insurance systems into a single-payer program that provides comprehensive healthcare to nearly 99% of the population in Taiwan. Through this system, patients can choose their preferred physicians and health facilities, and they have the benefits of comprehensive population coverage, short waiting times, relatively low outpatient copayments, and use of the NHIRD for planning, monitoring, and evaluating healthcare services. However, the system has some disadvantages, including short consultation time, poor gate-keeping of specialist services, and lack of an effective referral system.

The NHI Bureau established a program to construct the NHIRD and to release claims data for academic and medical research. According to the regulations established by the Department of Health, patient identity is encrypted for confidentiality and security. These databases allow comprehensive utilization and provide enrollment information, including demographic data, outpatient visit records, hospitalization records, and drug prescription registry information, for all patients. Diseases are coded according to the 2001 International Classification of Diseases, Ninth Revision, Clinical Modification (ICD-9-CM). The accuracy of the diagnoses listed in the NHIRD has been validated previously for several diseases [18,19].

### Study sample

The institutional review board of Taipei Veterans General Hospital approved data collection efforts for this study. We used a special dataset, which included all migraine patients between 2004 and 2010 in Taiwan according to the ICD-9-CM migraine diagnosis codes (346.0×, 346.1×, and 346.9×). The total study population in our study was 749,027 patients diagnosed with migraine between Jan. 2005 and Dec. 2008. We identified all migraine patients who were newly prescribed with triptans between 2005 and 2008. The index date was defined as the date of the first triptan prescription. Patients had to be enrolled for a minimum of 12 months before the index triptan prescription and had to undergo a 24-month observation period after the index triptan prescription. The index triptans were defined as the triptans newly prescribed at the index date. Patients undergoing treatment with migraine prophylactic agents, including amitriptyline, propranolol, valproic acid, topiramate, and flunarizine, were to continue treatment with at least one of these agents for at least 28 days after the index date. We excluded subjects who were prescribed triptans within 1 year before the index date, were 20 years old or younger at the index date, or had a follow-up period of less than 2 years. All patients eligible for analysis were followed up for 2 years.

In Taiwan, three brands of triptans, i.e. index triptans, were available during the study period: oral sumatriptan (Imigran®, 50 mg), intranasal sumatriptan (Imigran Nasal Spray®, 20 mg), and oral rizatriptan (Migoff ®, 10 mg). Migraine-specific drugs were triptans and ergot alkaloids. Non-specific acute medications for migraine were defined as a prescription within the following four categories for a patient with a migraine diagnostic code: NSAIDs, opioids, salicylates, and acetaminophen.

### Definitions

Patients enrolled in the index triptan population were classified according to their refill patterns at the first refill within 2 years after the index date. The following categories were used for classification: (1) persistent users (continued to refill the index triptan prescription), (2) within-class switchers (switched from the index triptan to another triptan), (3) between-class switchers (switched from the index triptan to a non-triptan acute migraine agent), and (4) discontinuers (no further prescription of triptan or non-triptan acute migraine medications during the 2-year follow-up period). Patients were defined as having a “2-year retention of index triptan” when they refilled at least one more prescription of the index triptan within 90 days before the end of the 2-year follow-up period.

### Statistical analysis

Baseline demographic data included age, sex, income, and urbanization. Urbanization levels in Taiwan are divided into four strata according to the Taiwan National Health Research Institute publications; Level 1 designates the most urbanized areas, and level 4 designates the least urbanized areas. The Charlson Comorbidity Index score and cardiovascular disease risk equivalent were analyzed to determine overall systemic health. During the 2-year follow-up period, the overall numbers and percentages of persistence, switching, and discontinuation of index triptan were calculated. The probability of persistence at each refill was computed by dividing the number of patients with consecutive refills of the index triptan by the total number of patients in the index population. The probabilities of within- or between-class switches and discontinuations, as well as the persistence rate of the index triptan at the first refill within the 2-year follow-up period were similarly calculated. Additionally, we calculated the 2-year retention rate of the index triptan.

Univariate and multivariate, backward, conditional Cox proportional hazard models were used to identify the factors in association with continued use of triptans at the first refill and at the end of the 2-year follow-up period. Factors with a p-value < 0.1 in univariate analyses were entered into the multivariate analyses. Microsoft SQL Server 2012 (Microsoft Corp., Redmond, WA, USA) was used for data linkage, processing, and sampling. All statistical analyses were conducted with the STATA statistical software program (version 12.0; StataCorp., Texas, USA). A p-value < 0.05 was considered to be statistically significant.

## Results

### Characteristics of the study population

We identified 13,951 migraine patients who were newly prescribed with triptans between January 2005 and December 2008 and met the inclusion criteria. At the index date, patients had a mean age of 41.3 years (standard deviation, 13.7 years), and 77.0% of them were women. 8.5% of the participants had cardiovascular disease, 9.2% had diabetes mellitus, and 1.4% had peripheral vascular disease. As the index prescription, 55.8% were prescribed only one triptan, and 42.7% were prescribed one triptan and other specific or non-specific acute migraine medications. Only four patients were prescribed rizatriptan; the remaining patients were prescribed sumatriptan (99.9% of the cohort).

Overall, 9,397 patients (67.4%) were prescribed at least one kind of migraine prophylactic agent, including propranolol (43.0%), flunarizine (30.8%), topiramate (23.2%), valproic acid (6.3%), and amitriptyline (4.3%). Most triptans were prescribed in the medical center (46.9%) and by board-certified neurologists (67.4%). The detailed results are shown in Table 1.

**Table 1:** Baseline characteristics of patients using triptans (2005–2008)

	**Number (percentage)**
No. of patient	13951
Female	10736 (77.0)
Age, mean (SD), years	41.3 (13.7)
Monthly income NT$	
Dependent	2782 (19.9)
NT 0–19,200	3837 (27.5)
NT 19,200-42,000	5383 (38.6)
>NT42,000	1949 (14.0)
Urbanization	
1 (Most urbanized)	6639 (47.6)
2	6528 (46.8)
3	6741 (4.8)
4 (Least urbanized)	110 (0.8)
Level of hospital	
Level I (Medical center)	6548 (46.9)
Level II	4425 (31.7)
Level III	2066 (14.8)
Level IV (Local medical clinics)	912 (6.5)
Charlson comorbidity index score	
Score 0	6521 (46.7)
Score 1	3787 (27.2)
Score 2	1849 (13.3)
Score ≥3	1794 (12.9)
Cardiovascular disease risk equivalent	
Cerebrovascular disease	1187 (8.5)
Diabetes mellitus	1287 (9.2)
Peripheral vascular disease	201 (1.4)
Triptan prescription by a neurologist	9396 (67.4)
All ambulatory visits for any kind of disease within one year before, median (IQR)	20 (11–35)
All ambulatory visits for headache within one year before, median (IQR)	1 (0–5)
Neurologic ambulatory visits within one year before, median (IQR)	0 (0–2)
Neurologic ambulatory visits for headache within one year before, median (IQR)	0 (0–1)
Triptan only	7784 (55.8)
Triptan plus other acute medications	6167 (44.2)
Sumatriptan, oral	13841 (99.2)
Sumatriptan, nasal	105 (0.75)
Sumatriptan, oral + nasal	1 (0.01)
Rizatriptan	4 (0.03)
Prophylactic agents	
Amitriptyline	594 (4.3)
Propranolol	5995 (43.0)
Valproic acid	884 (6.3)
Topiramate	3237 (23.2)
Flunarizine	4294 (30.8)
At least one prophylactic agent	9397(67.4)

1 US dollar = 29.9 New Taiwan Dollar (NT) on 1/1/2014.

### Triptan prescriptions during the 2-year follow-up period

At the first refill within the 2-year follow-up, 34.3% of patients refilled their index triptan prescription at least once (i.e., persistent users), 40.9% were between-class switchers, and 0.01% were within-class switchers (i.e., switched to different triptans). Notably, 24.8% did not seek any acute migraine treatment (i.e., discontinuers). Table 2 depicts the prescription patterns among between-class switchers (n = 5,705) at the first refill. These users filled prescriptions for NSAIDs (45.9%), acetaminophen (45.7%), ergotamine (27.7%), opioids (3.0%), and salicylates (2.1%). The rates of persistence, switching, and discontinuation at each refill are presented in Table 3. After the fourth refill, 10% or less of the index population remained adherent to the index triptan. At 2 years after the index date, only 4.0% of patients persistently used the index triptan (i.e., rate of refill retention at 2 years = 4.0%).

**Table 2:** Prescription patterns among between-class switchers at the first refill

	**N**	**(%)**
Switched to non-triptan	5,705	100.00
Salicylates	118	2.07
Acetaminophen	2,608	45.71
Opioids	171	3.00
NSAIDs	2,616	45.85
Ergotamine	1,581	27.71
Opioids and salicylates	2	0.04
Opioids and NSAIDs	13	0.27
Opioids and ergotamine	43	0.75

*Abbreviations:NSAIDs* non-steroidal anti-inflammatory drugs.

**Table 3:** Persistence, switching, and discontinuation at each refill (N = 13,951), % of total cohort

	**N**	**Within-class switchers**	**Between-class switchers**	**Discontinuers**	**% Persistent users**	**% Within-class switchers**	**% Between-class switchers**	**% Discontinuers**
Index	13951							
1st refill	4787	1	5705	3458	34.31	0.01	40.89	24.79
2nd refill	2775	0	1206	807	19.89	0	8.64	5.78
3rd refill	1922	0	477	376	13.78	0	3.42	2.70
4th refill	1457	0	279	186	10.44	0	2.00	1.33
5th refill	1148	0	181	128	8.23	0	1.30	0.92
6th refill	916	0	128	104	6.57	0	0.92	0.75
7th refill	780	0	63	73	5.59	0	0.45	0.52
8th refill	648	0	44	88	4.64	0	0.32	0.63
9th refill	540	0	43	65	3.87	0	0.31	0.47
10th refill	453	0	20	67	3.25	0	0.14	0.48
11th refill	396	0	15	42	2.84	0	0.11	0.30
At 2 years	13951	1	8161	5394	4.00	0.01	58.50	38.7

### Factors associated with persistent triptan use at the first refill

Univariate analysis revealed an increased risk of adherence to index triptans at the first refill in migraine patients with age of 40 years or greater; residence in urbanization levels 2, 3, and 4; monthly income of NT$ 19,200 or greater; first prescription by a neurologist; use of prophylactic agents; or frequent ambulatory visits for headache. Risk of adherence to triptans was reduced in patients with diabetes mellitus, cerebrovascular diseases, or first prescription at a local medical clinic (Table 4).

**Table 4:** Factors associated with continuing using index triptan at first refill

	**Univariate**		**Multivariate**	
**Variable**	**Odds ratio (95 CI)**	**p value**	**Odds ratio (95 CI)**	**p value**
Age				
20-40	1			
≥40	0.88 (0.82-0.94)	<0.001		
Male	0.97 (0.89-1.05)	0.461		
Urbanization				
Level 1 (Most urbanized)	1			
Level 2	0.91 (0.85-0.98)	0.012		
Level 3	0.77 (0.65-0.92)	0.003		
Level 4 (Least urbanized)	0.70 (0.47-1.06)	0.096		
Monthly income (NT$)				
Dependent	1			
NT$ 0–19,200	1.05 (0.94-1.16)	0.377		
NT$ 19,200-42,000	1.16 (1.05-1.28)	0.003		
>NT$ 42,000	1.46 (1.29-1.64)	<0.001		
Diabetes mellitus	0.62 (0.54-0.71)	<0.001	0.75 (0.65-0.87)	<0.001
Cerebrovascular diseases	0.77 (0.68-0.88)	<0.001		
Peripheral vascular disease	0.79 (0.58-1.08)	0.136		
Frequency of ambulatory visit for headache within one year before index date	1.02 (1.02-1.03)	<0.001	1.01 (1.00-1.02)	0.012
Neurologic ambulatory visit for headache within one year before index date	1.09 (1.08-1.11)	<0.001	1.05 (1.04-1.07)	<0.001
First prescription including triptan and other acute medications	1.35 (1.26-1.45)	<0.001	1.09 (1.01-1.17)	<0.001
First prescription of triptan by neurologists	3.45 (3.17-3.77)	<0.001	3.08 (2.81-3.37)	<0.001
Use of at least one prophylactic agent*	1.46 (1.35-1.58)	<0.001	1.37 (1.27-1.49)	<0.001
Hospital level of first prescription of triptan				
Level I (Medical center)	1		1	
Level II	0.95 (0.87-1.03)	0.205	0.97(0.89-1.06)	0.494
Level III	0.98 (0.88-1.09)	0.719	1.02 (0.91-1.14)	0.717
Level IV (Local medical clinics)	0.79 (0.68-0.92)	0.002	0.74 (0.63-0.87)	<0.001

*Prophylactic agents include amitriptyline, propranolol, valproic acid, topiramate and flunarizine.

In multivariate analysis, increased frequency of neurologic ambulatory visits for headache within 1 year before the index date, first prescription of triptan by a neurologist, first prescription of triptan and other acute medications, and use of at least one migraine prophylactic agent were associated with a significantly increased probability of continuing index triptan use at the time of the first refill. In contrast, diabetes mellitus and first prescription of triptan in a local medical clinic were significantly associated with a reduced probability of continuing use of the index triptan at the time of the first refill (Table 4).

### Factors associated with persistent triptan use at 2 years

Univariate analysis revealed an increased risk of adherence to index triptans at 2 years in migraine patients with a monthly income of NT$ 42,000 or greater, first prescription by a neurologist, or increased frequency of neurologic ambulatory visits for headache. In contrast, patients with diabetes mellitus or first prescription at a local medical clinic had a reduced risk of adherence (Table 5).

**Table 5:** Factors associated with retention refill of index triptan at 2 years

	**Univariate**		**Multivariate**	
**Variable**	**Odds ratio (95 CI)**	**p value**	**Odds ratio (95 CI)**	**p value**
Age				
20-40	1			
≥40	1.06 (0.89-1.26)	0.509		
Male	1.15 (0.94-1.39)	0.170		
Urbanization				
Level 1 (Most urbanized)	1			
Level 2	0.93 (0.78-1.11)	0.425		
Level 3	0.63 (0.39-1.02)	0.061		
Level 4 (Least urbanized)	1.05 (0.43-2.60)	0.909		
Monthly income (NT$)				
Dependent	1			
NT$ 0–19,200	0.80 (0.61-1.07)	0.131		
NT$ 19,200-42,000	1.22 (0.96-1.56)	0.108		
>NT$ 42,000	2.12 (1.62-2.77)	<0.001		
Diabetes mellitus	0.47 (0.32-0.71)	<0.001	0.59 (0.39-0.89)	0.011
Cerebrovascular diseases	0.74 (0.52-1.04)	0.085		
Peripheral vascular disease	0.87 (0.41-1.86)	0.721		
Frequency of ambulatory visit for headache within one year before index date	0.99 (0.99-1.01)	0.892		
Neurologic ambulatory visit for headache within one year before index date	1.04 (1.02-1.07)	0.001	1.05 (1.02-1.07)	0.002
First prescription including triptans and other acute medications	1.07 (0.90-1.27)	0.427		
First prescription of triptan by neurologists	3.02 (2.37-3.84)	<0.001	2.70 (2.12-3.45)	<0.001
Use of at least one prophylactic agent*	1.03 (0.86-1.24)	0.722		
Hospital level of first prescription of triptan				
Level I (Medical center)	1		1	
Level II	0.71 (0.58-0.86)	0.001	0.74 (0.60-0.90)	0.003
Level III	0.86 (0.68-1.11)	0.247	0.91 (0.71-1.17)	0.476
Level IV (Local medical clinics)	0.36 (0.22-0.61)	<0.001	0.34 (0.20-0.58)	<0.001

*Prophylactic agents include amitriptyline, propranolol, valproic acid, topiramate and flunarizine.

In multivariate analysis, increased frequency of neurologic ambulatory visits for headache within 1 year before the index date and first prescriptions of triptan by a neurologist were associated with a significantly increased probability of continuing index triptan use at 2 years. Diabetes mellitus and first prescription of triptan at a local medical clinic were significantly associated with a reduced probability of continuing index triptan use at 2 years (Table 5).

## Discussion

The results of our nationwide cohort study demonstrate that most first-time triptan users are females aged 30–50 years and prescribed triptans by a neurologist. The index triptan prescriptions were refilled by 34.3% of patients, whereas about 40% switched to non-triptan acute migraine agents at the first refill. Due to limited availability of different triptans in Taiwan, only 0.01% refilled their prescriptions with an alternative triptan. Overall, the rate of refilling triptans decreased markedly after the first refill. At the end of the 2-year follow-up period, only 4.0% maintained their index triptan.

### Comparison with US and European studies

Table 6 compares triptan refill patterns at the first refill and after 2 years among five cohorts (i.e., for this study and in the United States and Europe). We adopted a similar methodology to those applied in retrospective cohort studies of a pharmacy claims database in the United States and nationwide practice databases in Europe [16,17].

**Table 6:** Refill patterns at the first refill and 2-year retention, % of total cohort

	**Taiwan**	**US**	**UK**	**France**	**Germany**
Persistent users	34.3	25.5	44.3	34.2	36.7
Within-class switchers	0.01	7.4	4.9	6.8	6.3
Between-class switchers	40.9	67.1	2.3	4.0	2.3
Opioids	1.2	18.2	0.1	0	0
NSAIDs	18.8	12.5	0.9	2.0	1.3
Barbiturates	0	2.6	0	0	0
Ergot derivatives	11.3	0	0.2	0.6	0.1
Discontinuers	24.8	25.5	48.5	54.9	54.7
2-year retention rate	4.0	6.4	13.0	6.0	9.0

*Abbreviations:NSAIDs* non-steroidal anti-inflammatory drugs.

Among the five cohorts, at the time of the first refill, 25–45% of triptan users continued the index triptan, 53–66% discontinued the index triptan, and 25–55% stopped all acute migraine medications [16,17]. Approximately 5–7% of US and European triptan users switched to alternative triptans, compared to only 0.01% in Taiwan. The proportion of between-class switchers was higher in Taiwan and the United States than in Europe [17,16]. Most between-class switchers in the United States switched to opioids or barbiturates, whereas they primarily shifted to NSAIDs or ergot derivatives in Taiwan [16,17]. At the end of the 2-year follow-up period, only 4–13% retained their index triptan [16,17].

Although the refill pattern was similar among different countries, there were discrepancies related to the accessibility of abortive medications in the different healthcare systems. The accessibility of triptans is limited in Taiwan, and barbiturates are not available in Taiwan or Europe. Unlike many Western countries, after implementation of the NHI in Taiwan, the medical treatment-seeking behavior of patients changed because of freedom to select any healthcare provider or hospital based on preference without referral; on average, a person had 15.1 physician consultations per year in 2013 [20]. In Taiwan, triptans can be prescribed by a neurologist or primary care physician, as long as the indication fulfills the regulations recommended by the NHI.

### Factors associated with persistence and discontinuation

Patients with higher frequencies of clinic visits for headache before the index date might have experienced more severe headaches, which could have precluded their premature termination of treatment. In a previous study, patients reported fewer side effects of triptans with increasing age and patients who tolerated triptans better were more likely to continue triptans [21].

Prescription by a neurologist was a contributing factor for sustained triptan use. Patients who visit a specialist may be more confident in their prescriber or receive more detailed information about triptan use [22]. Confidence in specialists and satisfaction with the ability of triptans to eliminate migraine and restore normal functioning are important predictors of adherence to triptans [23]. Although migraine prophylaxis does not provide a cure, the effectiveness of abortive treatments is usually increased when combined with prophylactic drugs [24].

Triptan discontinuation has been associated with concomitant narcotic use, medication overuse, and higher Migraine Disability Assessment Test or Beck Depression Inventory scores [25,22]. When selecting an oral triptan, migraine sufferers and primary care physicians consider triptan effectiveness more important than tolerability of side effects or consistency of triptan effects [26]. Moreover, the patient’s desire to play an active role as a decision-maker should not be overlooked and insight is needed into individual needs and whether expectations are being fulfilled [27].

Considering the side effects, drug interactions, and contraindications of triptans, it may be that medical comorbidities, especially diseases with cardiovascular disease risk, influence the adherence to triptans. Migraine patients with diabetes mellitus tended to discontinue triptan, although we cannot provide valid explanations for this phenomenon. The other significant factor associated with triptan discontinuation was triptan prescription by a physician at a local clinic or community hospital. This result can be attributed to the fact that patients in Taiwan are less confident in the medical services provided at local medical institutes.

### Reasons for low percentage and frequency of triptan use

In an earlier study, migraine patients tended to discontinue triptans immediately after the index date [16]. Potential reasons for low triptan refills may include the side effects of triptans, lack of efficacy, uncertain diagnosis of headache, and diminished headache frequency or headache improvement.

In Taiwan, 32.5% of neurologists, especially those in solo practice, never prescribe triptans for migraine because of their high cost [28]. Concurrent use of non-triptan medications was fairly common in sustained triptan users to abort acute migraine attacks [16]. Prescription data reveal a very low percentage (0.4–1.4% of the general population) of triptan use in several countries; this circumstance was common and universal among migraine patients [29-31].

More than 30% of patients and up to 40% of migraine attacks fail to respond to a particular triptan, either because of suboptimal efficacy or tolerability issues [32]. Although patients who respond poorly to one triptan may respond better to another, most patients discontinue triptan treatment without trying another kind [33]. For many patients, satisfactory responses to over-the-counter (OTC) drugs and NSAIDs, as well as concomitant use of prophylactic agents, may impede the need to seek for medical help and further prescription of triptans.

## Conclusion

The tendency to discontinue triptan treatment after the initial prescription among migraine patients appears to be a universal finding across countries. Reasons for triptan non-adherence and relationships among demographic, psychological, and behavioral correlates and triptan adherence warrant further investigation.

### Potential study limitations

The data analyzed in our cohort study were obtained from the NHIRD of Taiwan. The NHIRD only records NHI-covered medical system usage. Therefore, data regarding the use of drugs not covered by the NHI, such as OTC drugs, were not available for study. Although these disadvantages should be accounted for or minimized through the study design, the NHIRD can be considered as a powerful tool for pharmaco-epidemiology studies. Lack of information on adverse events in the database makes it impossible to analyze the relationship between adherence/persistence and triptan tolerability. Because of the different healthcare systems adopted by Taiwan, the United States, and European countries, as well as the inherent limitations associated with the database study design, direct comparisons of the persistence and refill patterns of triptans should be interpreted with caution.

## Competing interests

The authors declare that they have no competing interests.

## Authors’ contributions

SJW conceptualized and designed the study, analyzed the data, and wrote the manuscript. TBC conceptualized and designed the study, and wrote the manuscript. YTC conceptualized, analyzed the data, and wrote the manuscript. JLF conceptualized, designed the study, and wrote the manuscript. CHT conceptualized, designed the study and analyzed the data. All authors read and approved the final manuscript.
